# Drivers of nocturnal and diurnal pollinating insect declines in urban landscapes

**DOI:** 10.1098/rspb.2025.0102

**Published:** 2025-08-06

**Authors:** Emilie E. Ellis, Stuart A. Campbell, Jill L. Edmondson

**Affiliations:** ^1^ School of Bioscience, The University of Sheffield, Sheffield, England, UK; ^2^ Research Centre for Ecological Change, Organismal and Evolutionary Research Programme, Faculty of Biological and Environmental Sciences, University of Helsinki, Helsinki, Finland

**Keywords:** urbanization, pollinator communities, Lepidoptera, Hymenoptera, Syrphidae, pollinator declines

## Abstract

Insect pollinators are essential for terrestrial ecosystems, delivering key ecosystem functions in the face of anthropogenic disturbance. Urbanization may be a key threat to pollinator communities. However, the scale of the threat remains unknown due to an overwhelming research emphasis on bees and a lack of comparative studies on hyper-diverse pollinating taxa such as nocturnal moths. As a result, it remains unclear which pollinator groups are most vulnerable to urbanization, and which habitat features are most critical for supporting them. We conducted a large-scale assessment of the effects of increasing urbanization on the diversity of bees, hoverflies and nocturnal moths in urban horticultural sites (allotments) across three cities. We report up to a 43% reduction in species richness along urbanization gradients, suggesting that a wide range of pollinators are under threat in urban landscapes. We show that these declines are driven by taxon-specific landscape drivers such as the reduction of tree canopy and semi-natural habitat, suggesting that urban insect conservation depends on the preservation or expansion of habitat features specific to different threatened taxa. We found that relative to bees, moths and hoverflies are particularly sensitive to urbanization, and we highlight the importance of including these frequently overlooked pollinator groups when assessing the biodiversity impacts of environmental change.

## 1. Introduction

Pollinating insects are vital for the reproduction of 60–90% of flowering plant species worldwide and have been deemed critical for 35% of crop species [1–3]. Conserving pollinator species diversity is essential for ensuring resilient pollination service delivery, particularly considering anthropogenic disturbance [4]. Declines in the abundance and diversity of all three major pollinating insect orders, including Hymenoptera (bees and wasps [5]), Diptera (flies [6]) and Lepidoptera (butterflies and moths [7–9]), are raising concerns about the fragility of pollination services that underpin the functioning of natural and managed ecosystems [4]. However, even for well-studied functional groups such as pollinators, the status of ‘less charismatic’ taxa (e.g. flies and nocturnal moths) remains poorly quantified. These data-deficient taxa may be disproportionately threatened by major anthropogenic forces due to the diversity of non-floral resource requirements needed to sustain their communities [10], making it important to understand their responses to these drivers at scale.

Urbanization causes habitat loss, fragmentation and disturbance [11] and is a key driver of insect declines [12,13]. However, cities are heterogeneous systems, characterized by a complex matrix of human-made impervious surfaces and greenspaces (e.g. gardens, parks, allotments) that vary in their management and the quality of ecological habitat they provide [14]. This landscape variation is often distributed along urban gradients, with higher proportions of infrastructure and impervious surfaces in urban centres compared with suburban areas, which have higher proportions of greenspaces interspersed within the built landscape. Landscape composition across cities can affect insect community structure (e.g. [15,16]); however, these effects are highly dependent on the intensity and heterogeneity of land use and land cover, the spatial scale examined and the taxonomic groups studied, and few comparative studies have simultaneously considered these factors.

Pollinator taxa are predicted to vary strongly in their responses to urban landscape features because of ecological filtering, which selects for specific traits that allow some species to persist in cities [17,18]. For example, pollinators active at night (i.e. nocturnal moths) are extremely sensitive to the negative effects of light pollution on their reproduction [19], diapause [20] and foraging [21,22]. Both diurnal and nocturnal pollinators face limited habitat availability, and this may be particularly important for taxa with complex life-history traits. For example, many hoverfly species are dependent on stagnant freshwater and insect prey to complete their life cycles, which can be rare in urban systems [23]. Conversely, bees are dependent on high floral availability in urban greenspaces, particularly pollen for larval provisioning, and therefore can use the high floral density in urban greenspaces. Their comparatively simple ecological niches may explain why bees may be more persistent in urban areas compared with hoverflies [15,16]. The major pollinating orders (Diptera, Hymenoptera and Lepidoptera) play complementary roles in the pollination of urban plant communities [21], and considering how urbanization affects all these pollinator taxa is therefore essential to ensure the stability and resilience of pollination in urban ecosystems.

Numerous studies have documented the effects of urbanization on individual pollinator taxa (e.g. bees [24], hoverflies [25] and moths [26]). However, comparative studies that provide an essential window into the relative risks to different taxa are lacking, particularly studies investigating the relative sensitivity of diurnal (bee and hoverfly) and nocturnal (moth) pollinating insect communities to urbanization (but see [27]). Diurnal and nocturnal pollinating insects coexist within the same ecological system, synergistically fulfilling the essential functional role of pollination. However, variation in niche breadth among taxa suggests that fly and particularly moth taxa may be disproportionately affected by urbanization compared with bees. This variation in sensitivity is likely to be driven by different habitat features within urban greenspaces. Greenspaces vary significantly in plant species composition, floral density and vegetation structure [14], and these differences are likely to have unique effects on different insect taxa. For instance, bees have been shown to benefit from higher floral density (e.g. [24]), whereas moths require sheltered habitat (e.g. shrubs and trees: [21,28,29]). However, these taxon-specific responses to different urban habitat features have yet to be tested.

Here, we test the hypothesis that major groups of pollinating insects differ in their responses to urbanization as a result of taxon-specific habitat requirements. Focusing on a broad range of diurnal and nocturnal pollinating insects including bees, hoverflies and moths, we provide a direct comparison of how features of urban landscapes are affecting the relative abundance and diversity of pollinators in three UK cities (Leeds, Leicester and Sheffield). Landscape-level habitat data (impervious surface, semi-natural habitat, gardens and area of tree canopy cover) were then used to test the mechanisms driving changes in pollinator community composition within and across pollinating insect taxa.

## 2. Material and methods

### (a) Study area description

**Figure 1.**
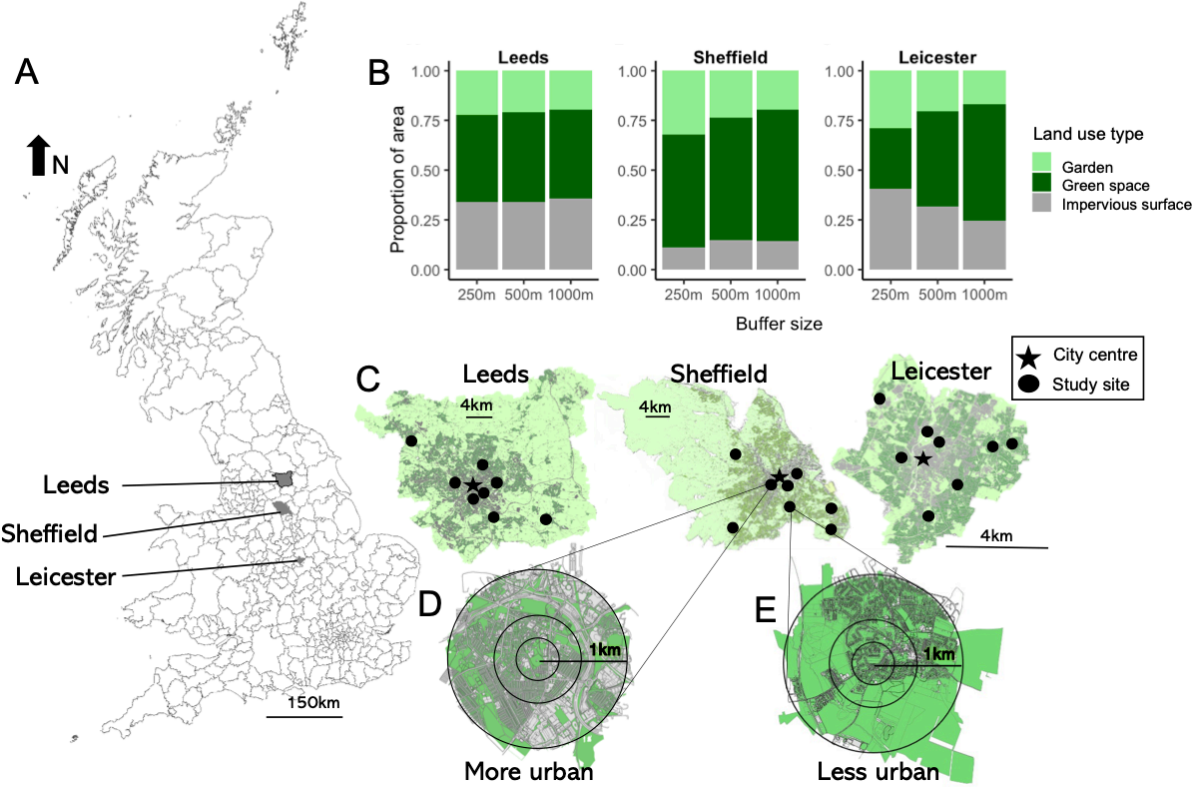
Landscape composition of Leeds, Sheffield and Leicester. (A) The city locations within the UK. (B) The proportion of area of garden, greenspace and impervious cover surrounding eight allotment sites in each city (Leeds, Sheffield and Leicester), at three different scales surrounding the site. Circular buffers with radii of 250, 500 and 1000 m were drawn around the sites. (C) Maps of Leeds, Sheffield and Leicester showing the site locations and the distribution of greenspace (green) and impervious cover (grey) areas. (D) An example of an allotment with a high density of surrounding impervious surfaces and (E) an example of an allotment with a low density of surrounding impervious surfaces. Circles within D and E depict the circular buffers at which landscape composition was measured: 250, 500 and 1000 m.

Three UK cities were selected for this study, Leeds, Leicester and Sheffield, which vary in size and urban intensity (figure 1, table 1). Our cities were chosen to capture the variation of urban intensities across UK cities. Within each city, eight allotment sites were selected along a gradient of urbanization from the city centre to the edge of the administrative boundary (electronic supplementary material, figure S1). Allotments are relatively large greenspaces used for urban horticultural production. Each allotment site contains a number of allotment plots (approx. 250 m^2^) rented to an individual or household to grow fruit and vegetable crops [30]. Distance from the city centre was a good proxy for increasing urbanization, explaining over two-thirds of the variation in impervious cover surrounding our sites (at a 1000 m buffer; Adj *R*^2^ = 0.72, *p* = 0.001; electronic supplementary material, figure S2). Within Sheffield and Leeds, the eight selected sites were at least 2 km apart; however, Leicester is only a small city (only 14 km wide) and so had some sites that were closer together (minimum distance 1 km). Although the dispersal abilities of some insect taxa are greater than the distance between sites, the disturbed nature of urban environments, such as habitat fragmentation and topographic barriers (e.g. buildings), means our sites are likely to be largely independent.

**Table 1.** Summary table of the properties of three cities, Leeds, Leicester and Sheffield, and the pollinators collected in them. On the left: city properties (total area (km²) of the city, total area of impervious surfaces (km²) and the proportion (%) of impervious surface). On the right, the abundance and species richness of bees, hoverflies and moths collected in allotments between May and October 2019 in each city (eight sites per city).

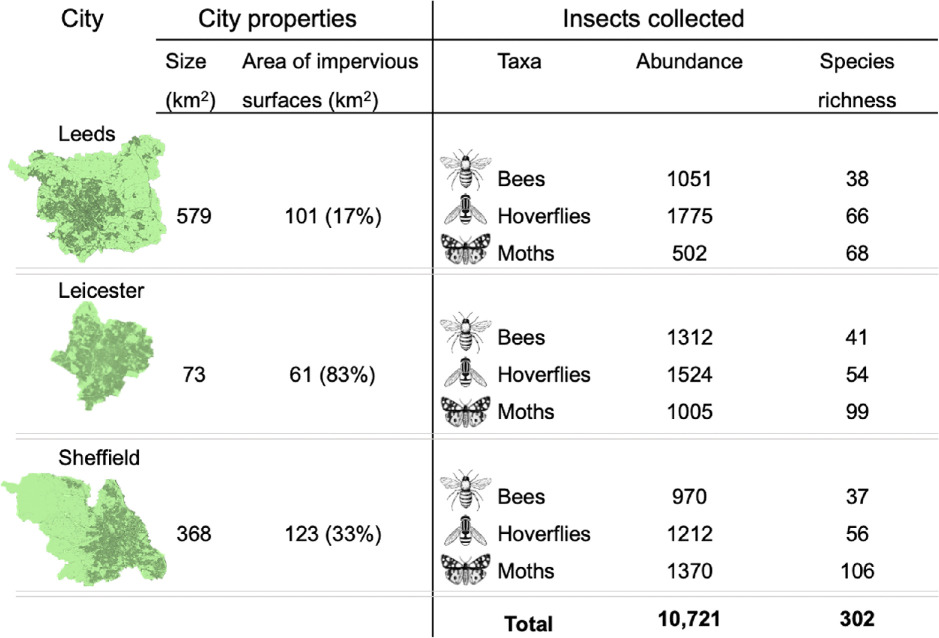

### (b) Pollinator sampling

We measured the abundance and species richness of representative groups in three orders of flying insect pollinators: diurnal hoverflies (Diptera: Syrphidae), bees (Hymenoptera: Anthophila) and nocturnal moths (Lepidoptera). We sampled pollinators for six months (May–October 2019) using a combination of biodiversity monitoring methods, including Malaise traps, sweep netting and light trapping. The combination of techniques may not capture all potential pollinators but will enhance the overall robustness of the study through a synergistic effect: each method has specific strengths, and when used together, they compensate for not all but many potential biases arising from using single techniques. For example, Malaise traps are passive and do not target specific insects but can be less effective at collecting large-bodied flying insects; however, they allow standardized sampling, particularly across periods of variable weather. Conversely, sweep netting has a larger user bias as the insects are targeted by the collector, but they are effective at collecting large flying insects.

#### (i) Malaise traps

These flight intercept traps are time- and cost-effective and have been shown to be well suited for comparative studies of the flying arthropod fauna at a large geographical scale [31]. Twenty-four Malaise traps (NHBS product code 262550) (one per site) were simultaneously deployed for the first seven days of each month from May to October 2019. The Malaise traps were set up close to the centre of the allotment along a natural insect flight path (i.e. perpendicular to the main path through the allotment). The sample bottles were filled one-third with 100% non-toxic propylene glycol. All bees and hoverflies were sorted from the samples and identified.

#### (ii) Timed transects

Diurnal bees and hoverflies were collected at each site at three time points during the season (early summer = May; midsummer = June; late summer = September) by timed line transects through the main path of the site and also on individually managed plots. Site transects were carried out for a fixed time of 30 min along the main path running through the allotment between 9.00 and 17.00 on clear, calm, warm days. For plot-level transects, three individually managed plots (chosen randomly across the site) were sampled for 10 min per plot. All bees and hoverflies visiting flowers along the transects were collected using a hand net and identified.

#### (iii) Light traps

Nocturnal moths were sampled at the same time as the transect collections (early summer = May; midsummer = June; late summer = September) on calm, warm nights using a 12 volt portable Heath Trap equipped with a 15 W actinic bulb (NHBS product code SK22). All sites within each city (*n* = 8) were sampled from dusk until dawn on the same night, and all cities were sampled within five days of each other at each time point.

### (c) Landscape variables

To determine the main environmental drivers shaping pollinator biodiversity along an urbanization gradient, we gathered a series of landscape-level variables at multiple scales. Observing scale dependency is not unusual in urban studies (e.g. [32]). Analysing a single scale might lack either the variability needed to detect true responses or present unique conditions by chance, leading to strong but potentially inaccurate conclusions. By employing multiple scales, we can more accurately test and confirm consistent ecological patterns, thus enhancing the reliability and power of our findings. Therefore, we quantified landscape variables at three spatial scales surrounding each site: circular buffers with radii of 250, 500 and 1000 m were drawn using ArcGIS. Land cover in each buffer was extracted from OS Mastermap, and tree canopy cover was extracted from the National Tree Map (Bluesky ©). The land cover characteristics we extracted at a landscape scale were (i) the area of impervious surfaces (buildings, roads and impervious surfaces), (ii) the area of semi-natural cover (scrub, grassland and shrubs), (iii) the area of gardens, and (iv) the area of tree canopy.

### (d) Data analysis

All analyses were done in R v. 4.12 [33].

### (e) Sample efficiency

We used the iNEXT package (iNEXT : : iNEXT [34]) to compute diversity estimates and explore how well our pollinator communities were sampled. Specifically, we used sample-size-based curves. We first estimated the sample efficiency of all pollinator sampling methods combined and then compared the sample efficiency of Malaise traps, sweep-netting and light trapping individually.

### (f) Model fitting and inference

Pollinator data were aggregated across the season, and pollinator communities were characterized for moths, bees and hoverflies at each site by abundance (total number of insects) and species richness (total number of insect species). These two variables were all subsequently analysed as the response variables in our models (outlined below).

We used linear mixed effect models (using lme4 : : lmer [35]) to understand how insect communities respond to changes in landscape composition while also assessing potential variations across different pollinator taxa and cities. To achieve this, we considered three taxa (bees, hoverflies and moths) and each city (Leeds, Leicester and Sheffield) as three-level factorial variables, along with their interactions with each other and the continuous habitat variables.

We found that environmental explanatory variables (area of impervious cover, semi-natural area, tree canopy and gardens) were frequently correlated (see correlation matrices in electronic supplementary material, figure S3). To assess how this collinearity might influence model fitting, we conducted a preliminary analysis using variance inflation factors (VIF), a standard approach for detecting multicollinearity in regression models [36]. A custom function based on car::vif [37] was used to calculate VIF values for mixed models. We fit full models at each spatial scale (250, 500 and 1000 m), each including all habitat variables. VIF values were consistently high (generally greater than 9; see electronic supplementary material, table S1), supporting the use of a single-variable modelling approach.

Our analytical approach therefore involved testing each habitat variable separately at each spatial scale. This resulted in three scale-specific sets of models (250, 500 and 1000 m), each including a single land-use variable (a total of 12 models for pollinator species richness and 12 models for pollinator abundance). This approach allowed us to examine the independent effects of each habitat type without the confounding influence of collinearity. All models followed the same structure,


(3.1) Measure of pollinator diversity∼pollinator taxon+city+habitat variable+[all two-way interactions]+(1|Site).

In this structure, the response variable is either pollinator abundance or pollinator species richness; *pollinator taxon* is a three-level factor (bees, hoverflies and moths); *city* is a three-level factor (Leeds, Leicester and Sheffield); and the *habitat variable* is a continuous measure of either impervious surface area, garden area, tree canopy or semi-natural habitat surrounding each site.

We initially fit each individual model in full (with all two-way interactions) and chose our final model using an Akaike information criterion (AIC) based model selection approach (MuMIn : : dredge [38]). Models were fit using a Gaussian distribution on log-transformed response variables as the data conformed well to assumptions of normality after log-transformation. The log-Gaussian approach allowed for interpretable multiplicative effects, consistent with the biological interpretation of our response. To ensure model assumptions were met, residuals were visually examined to assess homoscedasticity, normality and the presence of influential points or non-random patterns. Statistical significance of model terms was assessed using type II Wald chi-square tests (analysis of deviance, car : : Anova [37]). When there was significant interaction, we conducted post hoc tests using estimated marginal means (emmeans : : joint_test [39]). All *p*-values were adjusted using the false discovery rate method (stats : : p.adjust ‘fdr’ [33]) to account for multiple testing.

## 3. Results

### (a) Pollinator communities

Focusing on urban greenspaces used for horticulture (allotments), in three UK cities we used intensive sampling to characterize communities of key pollinating insect taxa (diurnal bees and hoverflies and nocturnal moths) and used land mapping to assess the surrounding landscape of each site at three scales (figure 1). Using monthly Malaise trap collections and transect walks throughout the growing season (May–September), we collected 10 721 insects belonging to 302 species. Hoverflies were the most abundant, accounting for 41% of all pollinators, and moths were the most species rich, accounting for 49% of the total species richness (table 1; species lists, electronic supplementary material, tables S2–S4). Domesticated honeybees, *Apis mellifera*, were highly abundant in our bee communities (25% of all bees recorded). Although honeybees are a managed species, they were retained in the analysis as they are an important component of the bee community and we found no qualitative differences in our results when they were excluded from the analysis (electronic supplementary material, figure S4).

### (b) Sample efficiency

Pollinator groups had similar sampling coverage: bees (mean coverage 62%), hoverflies (mean coverage 57%) and moths (mean coverage 50%). For diurnal sampling, we found that sweep nets and Malaise traps caught very different numbers of individuals: overall, Malaise traps caught 3793 hoverflies (84% of all hoverflies recorded), whereas sweep nets caught 2461 bees (73% of all bees recorded, electronic supplementary material, tables S5 and figure S5). We found that Malaise traps had mean sampling coverage of 59% for bees and 57% for hoverflies. Sweep-net samples had slightly higher sample coverage for both bees (78%) and hoverflies (65%), indicating that Malaise traps may be sampling more rare species (see full method comparisons in electronic supplementary material, table S5 and figures S5–S7). As the main goal of our inclusion of multiple methods was to minimize sampling biases related to method choice, we combined all data, and this is reported in all results below. Sampling method-specific analyses are reported in electronic supplementary material, figures S5–S7.

### (c) Urbanization and pollinator declines

**Figure 2.**
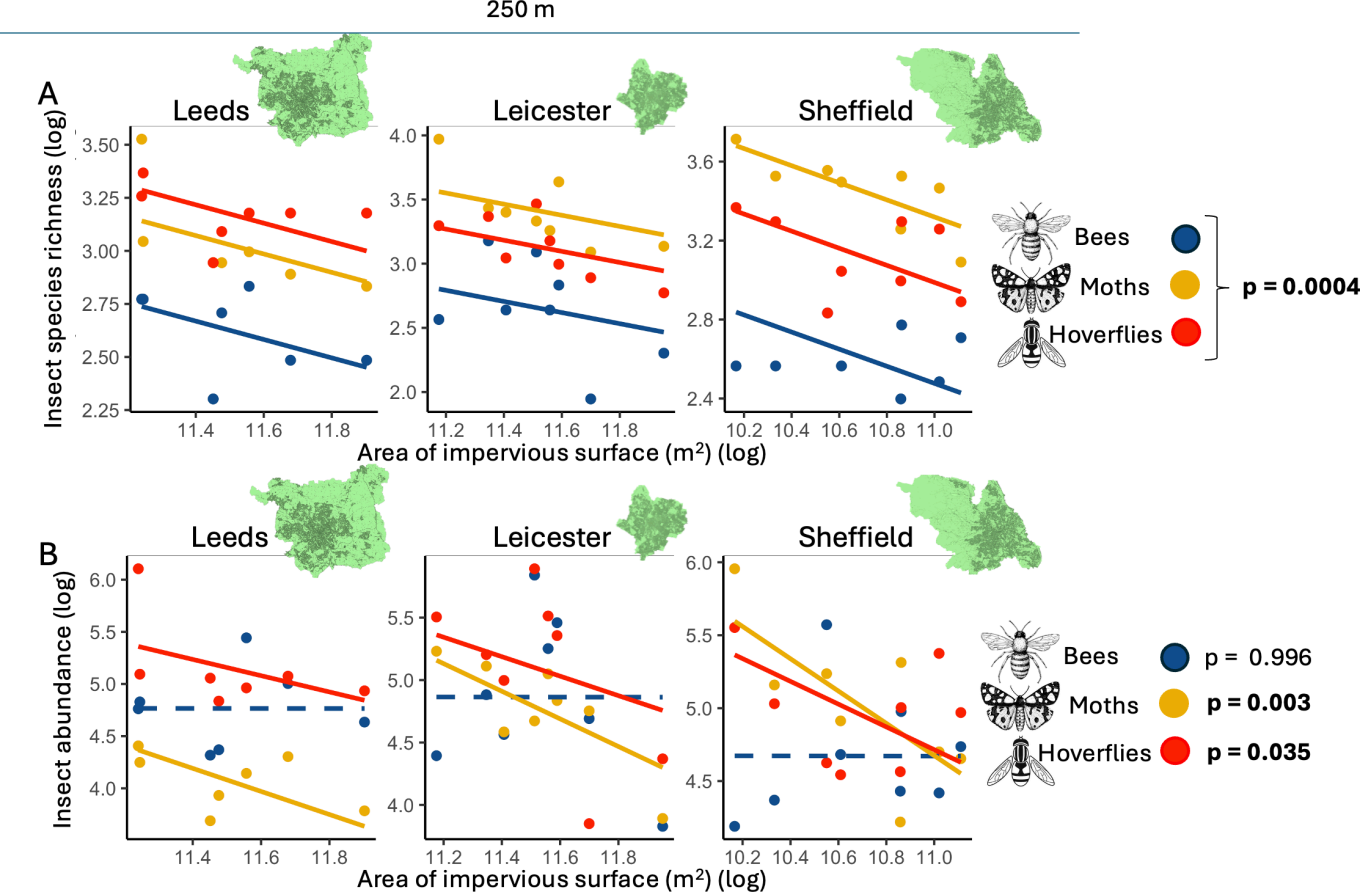
Plots showing (A) species richness and (B) abundance of moths (yellow), hoverflies (red) and bees (blue) in allotment sites (points) along gradients of increasing impervious surface (m^2^) in the area surrounding the site (at a 250 m buffer radius from the site centre). Lines illustrate linear mixed models testing how species richness and abundance change along increasing urbanization gradients across pollinator groups and cities (and their interactions where present). (A) Parallel lines show no significant pollinator taxon × urbanization interactions. (B) A significant pollinator taxon × urbanization interaction. Solid lines show significant effects (*p* < 0.05); dashed lines show non-significant effects (*p* > 0.05) derived from post hoc tests.

**Figure 3.**
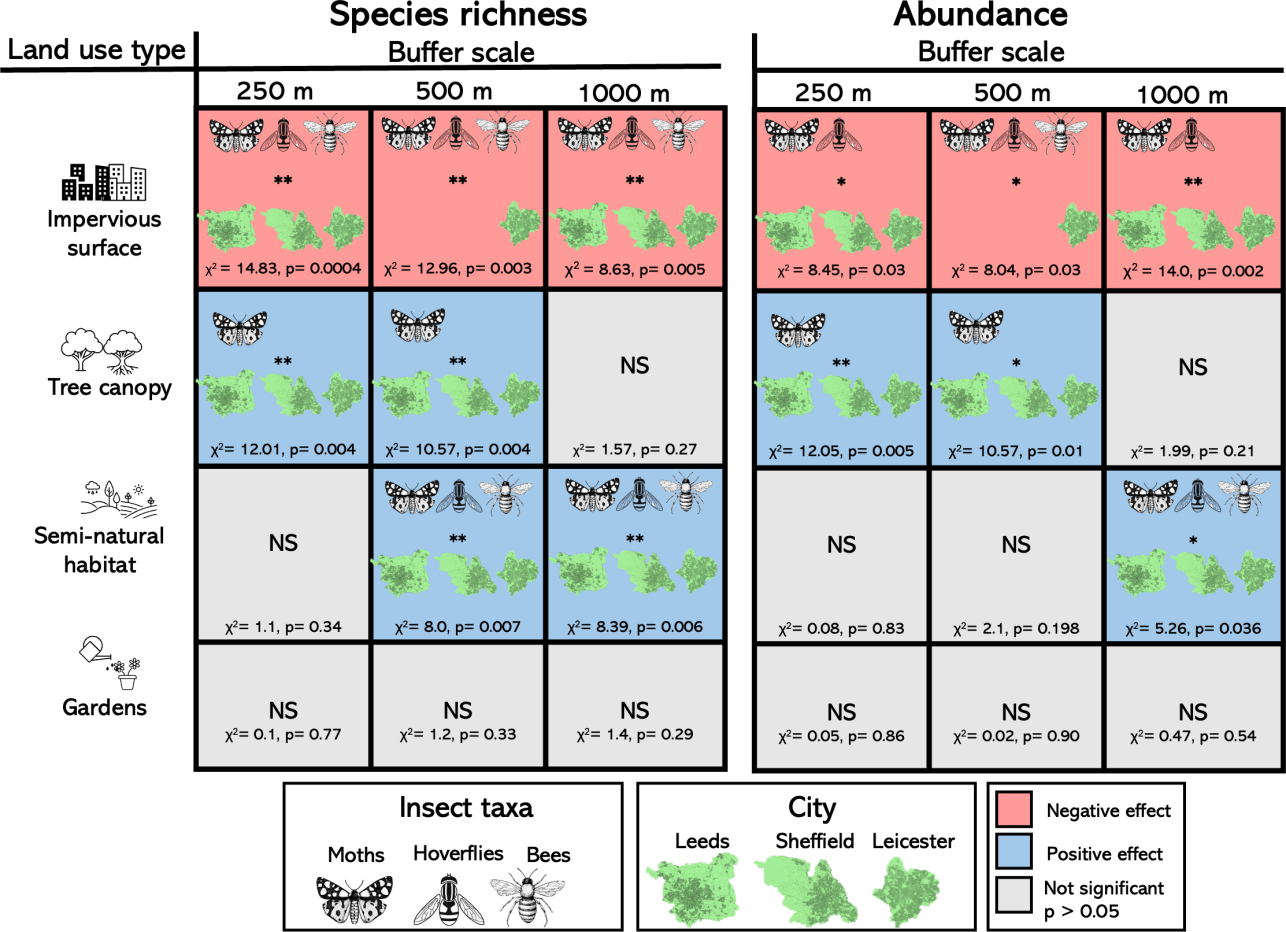
Landscape drivers of changes in pollinator communities in urban allotments across multiple scales. The effect of the area of impervious cover, area of tree canopy, area of semi-natural greenspaces and area of gardens on the species richness and abundance of bees, moths and hoverflies across the three cities. Landscape composition was measured at three scales surrounding sites of urban horticulture (250, 500 and 1000 m). Colours of cells indicate positive (blue) or negative (red) responses (or non-significant (grey), *p* > 0.05 ). All *p*-values are adjusted using false discovery rates to account for multiple testing. Statistics report a significant main effect when no interaction was significant. Icons of pollinator taxon and city indicate if there were significant interactions between the environmental variable and pollinator taxon or city. Full model outputs and *p*-value adjustments are found in electronic supplementary material, tables S6–S13.

Pollinator communities were negatively affected by increasing area of impervious surface, and this pattern was broadly congruent across all the three cities and for all three pollinator groups (figures 2 and 3, electronic supplementary material, tables S6 and S7). These declines were consistent despite differences in overall impervious surface and landscape composition across cities (figure 1). Although the species richness of all taxa declined at a comparable rate at all scales examined (no significant taxa × urbanization interaction), in absolute values, hoverflies and moths exhibited a twofold higher species loss due to urbanization compared with bees (figure 2). There was limited evidence that the pollinator responses to increasing urbanization were dependent on the spatial scale examined, with Leicester showing the greatest consistency across all three spatial scales (figure 3).

In contrast to the consistent effects on species richness, the effect of impervious surface on abundance differed depending on taxon (pollinator taxa × urbanization interaction figures 2B and 3). For example, at the 250 m scale, post hoc tests showed that the abundance of moths (*p* = 0.003) and hoverflies (*p* = 0.035), but not bees (*p* = 0.996), declined as urbanization increased (figure 2B, electronic supplementary material, table S7), and this pattern was also true at 1000 m (figure 3). At 500 m, there was a significant city × urbanization interaction (χ^2^ = 8.04, *p* = 0.03, figure 3), with post hoc tests revealing declines only in Leicester (electronic supplementary material, table S7).

### (d) Taxon-specific responses to greenspace habitat features

When greenspace composition (area of semi-natural habitat, gardens and tree canopy) was analysed across cities, the pollinator community responses varied with each habitat feature with evidence of taxon- and scale-dependent responses (figure 3, see full model summaries in electronic supplementary material, tables S8–S13). The area of tree canopy had significant positive effects on the species richness and abundance of moths only, and this was true for all cities. For species richness, the positive relationship was similar at 250 m (χ^2^ = 12.01, *p* = 0.004) and 500 m (χ^2^ = 10.57, *p* = 0.004) and was not significant at 1000 m (χ^2^ = 1.57, *p* = 0.27). For abundance, the relationship between canopy cover and moths showed similar patterns across scales (figure 3).

The area of semi-natural greenspace habitat was a constant predictor of increasing species richness of all three taxa across all three cities (figure 3). We found increases in species richness with increasing semi-natural habitat at 500 and 1000 m. Conversely, with abundance, we only found a detectable effect of semi-natural habitat at the 1000 m scale (figure 3).

In contrast to tree canopy and semi-natural habitat, the area of gardens had no detectable effect on the pollinator communities across all our analysis figure 3.

## 4. Discussion

Despite the distinct niches and life-history traits of diurnal bees, hoverflies and nocturnal moths, we observed comparable negative impacts of urbanization on the species richness of all three groups. Specifically, for every 10% increase in impervious surfaces, we saw a reduction of up to 7.5% in pollinating insect species richness for three major pollinating insect taxa. Our results demonstrate that increased impervious surfaces and associated declines in areas of semi-natural habitats and tree cover are the primary drivers of insect diversity in urban areas, underscoring the need to preserve or expand these habitats for pollinator conservation. Importantly, we do not find that the diversity of any specific group of pollinators is more robust to urbanization, in contrast to the findings by Merckx & van Dyck [27] and Piano *et al*. [40]. However, the causes of the reductions in the diversity of pollinators differed among taxa, highlighting the necessity of integrating overlooked insect groups such as moths into biodiversity assessments and sustainable urban planning.

While there were relatively similar declines of species richness in all taxa (figure 3), we found that pollinator abundance differed among pollinator groups, suggesting that moths and hoverflies may be disproportionately affected by urbanization compared with bees (figures 2B and 3). There are several potentially non-exclusive mechanisms for this variation in sensitivity based on divergence in life-history traits. First, bees use floral resources (nectar and pollen) as both adults and larvae [41], while most moths and hoverflies have distinct larval and adult resource requirements, often including specific host plants for foliar-feeding larvae [42] or specific larval prey and habitat requirements for hoverflies [23]. Thus, moths and hoverflies may be disproportionately affected by these added larval constraints. Second, the lower sensitivity of overall bee abundance to urbanization may be driven by social bees (*Apis* and *Bombus*), which accounted for 88% of bee abundance. Social bees possess numerous traits that may enable them to persist in urban environments, including social foraging, relatively large average foraging distances and a relatively wide range of suitable host plants (polylecty) [18]. These traits may confer greater ecological flexibility [17] compared with solitary bees, flies and moths. Finally, while both bees and moths may be limited by host plant diversity in urban centres [21] and are both negatively affected by urban air pollution [43,44], moths are also strongly affected by light pollution [22,45] making them more sensitive to urbanization compared with other taxa [40]. Our study suggests that trait diversity within and among the major groups of pollinating insects may be an important predictor of their relative sensitivity to urbanization.

The effect of urbanization on pollinating insect diversity across bees, hoverflies and moths carries significant implications for the stability of urban ecosystems. Conserving pollinator biodiversity can increase functional redundancy and buffer pollination against individual species losses [4]. This buffering effect is driven, in part, by the differences in plant visitation among taxa, particularly between diurnal and nocturnal pollinators. As such, the analysis of the pollen loads of moths and bees shows significant groups of plants that are only visited by one group or the other [21]. Although bees have been shown to be important pollinators, non-bee taxa provide important complementary pollination services. For example, flies and moths, though less studied, enhance the pollination of bee-visited flowers and can even be more efficient pollinators [2,46]. Divergence in behaviour can also lead to non-bee pollinators providing unique aspects to pollination. For example, hoverflies provide long-distance pollen transfer relative to bees [23], and nocturnal moths visit plants that only produce nectar at night [47]. Future studies should include other poorly understood taxa, such as non-syrphid flies, which may provide key pollination roles, as seen in Arctic regions [48]. Overall, our results highlight the power of comparative approaches to understanding how diverse taxa collectively accomplish pollination of urban plant communities.

The loss of 43% species richness along an increasing urbanization gradient raises additional concerns about the stability and functionality of urban ecosystems extending beyond pollination service delivery. For example, hoverfly larvae are important predators of crop pests such as aphids [49,50]. Like hoverflies, moths have non-floral resource requirements, and as herbivorous larvae, they act as pests but also as key drivers of other ecosystem functions such as nutrient cycling [51]. Larval and adult moths are also critical food sources for bats and birds [52–54] and underpin the stability of higher trophic levels and the biodiversity value of urban parks and woodlands. Our results thus suggest that a wider range of ecosystem functions associated with specific groups of pollinators are under comparable threat from urbanization.

Our simultaneous assessment of all three major pollinator groups in three cities allows us to disentangle some of the landscape and habitat characteristics that support or constrain urban pollinators and assess whether these differ for species with different traits or niches. There were taxon-specific responses to different urban greenspace habitats, and these were broadly consistent regardless of spatial scale (figure 3). For example, increased tree cover was a significant predictor of the diversity and abundance of moths, but not bees or hoverflies. Moths may benefit from the daytime roosting locations provided by tree structural complexity or from the use of trees as potential larval and/or adult food sources [21,26,29], suggesting that tree cover may be an important mitigating factor against the negative effects of urbanization for highly sensitive nocturnal taxa. Conversely, we show consistent positive effects of semi-natural habitat across all three taxa, indicating that these habitats may offer a wide range of resources for a wide range of taxa. These could include nesting sites for bees [55], prey for hoverflies [56], larval host plants and pupation sites for moths [57] and nectar and pollen sources for all insects [58]. Thus, our study suggests future work should aim to quantify the resources these semi-natural habitats provide for specific insect species.

One of the challenges of assessing drivers of urban insect declines is the high degree of inter-relatedness among the many landscape features which characterize urbanization, with potentially nonlinear impacts on different pollinators. While the habitat measures we examined were correlated, the consistent responses within, but divergent responses among, taxa suggest our analysis is detecting ecologically relevant drivers for the different pollinator groups. Our analysis also suggests that habitat features may have combined effects beyond habitat loss alone. For example, tree cover was not always highly correlated with impervious surface area, yet moth diversity consistently declined with increasing impervious surface and increased with greater tree canopy cover (figure 3), suggesting greater-than-additive benefits of increasing greenspace and tree cover for these taxa. Our study is focused on drivers of pollinator diversity in cities, but these results may have implications for understanding impacts of habitat loss in other human-modified landscapes. For example, compared with cities, agricultural landscapes differ in their effects on specific pollinator groups such as bees and flies [15,16,32], and future assessments of agricultural intensification may benefit from simultaneous comparison of multiple drivers and taxa.

Finally, a striking finding from our comparison of habitat drivers is the consistent lack of effect of the surrounding area of private gardens on any pollinator group, in contrast to prior studies of bees, hoverflies and moths [14,29,59]. Gardens comprised approximately 23.3% of our average greenspace in the three study cities. Urban domestic gardens vary significantly in their management, and the lack of effect of gardens in our analysis may be due to variation in grassy lawn area, amount of paving/tiling and the relative abundance of suitable and unsuitable vegetation (e.g. non-flowering hedges). Overall, our analysis suggests that communities of bees, but also moths and hoverflies, critically depend on the diverse resources provided by urban greenspaces. However, the quality and quantity of these habitats are the major drivers of how pollinators with divergent resource requirements can persist in urban environments.

## 5. Conclusion

Identifying the drivers of pollinator diversity declines is critical for protecting at-risk species and the roles in pollination they provide. Here, we show that increasing urbanization has negative effects on the species richness and abundance of both nocturnal and diurnal pollinators, including bees, moths and hoverflies. Our results show that aspects of urbanization pose both general and taxon-specific threats to different pollinators, suggesting that a more nuanced and mechanistic approach to pollinator conservation is required to protect pollinating insect communities. Accomplishing this goal will probably require appropriate urban planning and stakeholder engagement to sustain the specific habitat features required by pollinating insects.

## Data Availability

All data and code used in this manuscript is deposited in Dryad and is publicly available [60]. Supplementary material is available online [61].
